# A review of enhanced paramedic roles during and after hospital handover of stroke, myocardial infarction and trauma patients

**DOI:** 10.1186/s12873-017-0118-5

**Published:** 2017-02-23

**Authors:** Darren Flynn, Richard Francis, Shannon Robalino, Joanne Lally, Helen Snooks, Helen Rodgers, Graham McClelland, Gary A. Ford, Christopher Price

**Affiliations:** 1 Institute of Health and Society, Newcastle University, Baddiley-Clark Building, Richardson Road, Newcastle upon Tyne, NE2 4AX United Kingdom; 2 Institute of Neuroscience (Stroke Research Group), Newcastle University, Newcastle upon Tyne, United Kingdom; 3 Research Design Service - North East, Institute of Health and Society, Newcastle University, Newcastle upon Tyne, United Kingdom; 4 College of Medicine, Swansea University, Wales, United Kingdom; 5 North East Ambulance Service NHS Foundation Trust, Newcastle upon Tyne, United Kingdom; 6 Oxford University Hospitals NHS Foundation Trust, Oxford, United Kingdom

**Keywords:** Systematic review, Paramedics, Extended role, Stroke, Acute myocardial infarction, Trauma

## Abstract

**Background:**

Ambulance paramedics play a critical role expediting patient access to emergency treatments. Standardised handover communication frameworks have led to improvements in accuracy and speed of information transfer but their impact upon time-critical scenarios is unclear. Patient outcomes might be improved by paramedics staying for a limited time after handover to assist with shared patient care. We aimed to categorize and synthesise data from studies describing development/extension of the ambulance-based paramedic role during and after handover for time-critical conditions (trauma, stroke and myocardial infarction).

**Methods:**

We conducted an electronic search of published literature (Jan 1990 to Sep 2016) by applying a structured strategy to eight bibliographic databases. Two reviewers independently assessed eligible studies of paramedics, emergency medical (or ambulance) technicians that reported on the development, evaluation or implementation of (i) generic or specific structured handovers applied to trauma, stroke or myocardial infarction (MI) patients; or (ii) paramedic-initiated care processes at handover or post-handover clinical activity directly related to patient care in secondary care for trauma, stroke and MI. Eligible studies had to report changes in health outcomes.

**Results:**

We did not identify any studies that evaluated the health impact of an emergency ambulance paramedic intervention following arrival at hospital. A narrative review was undertaken of 36 studies shortlisted at the full text stage which reported data relevant to time-critical clinical scenarios on structured handover tools/protocols; protocols/enhanced paramedic skills to improve handover; or protocols/enhanced paramedic skills leading to a change in in-hospital transfer location. These studies reported that (i) enhanced paramedic skills (diagnosis, clinical decision making and administration of treatment) might supplement handover information; (ii) structured handover tools and feedback on handover performance can impact positively on paramedic behaviour during clinical communication; and (iii) additional roles of paramedics after arrival at hospital was limited to ‘direct transportation’ of patients to imaging/specialist care facilities.

**Conclusions:**

There is insufficient published evidence to make a recommendation regarding condition-specific handovers or extending the ambulance paramedic role across the secondary/tertiary care threshold to improve health outcomes. However, previous studies have reported non-clinical outcomes which suggest that structured handovers and enhanced paramedic actions after hospital arrival might be beneficial for time-critical conditions and further investigation is required.

## Background

Evidence-based care standards emphasise the importance of early recognition, appropriate conveyance and co-ordinated care for patients presenting as medical emergencies [1]. This is particularly important for optimising outcomes amongst those conditions with time-critical treatments, notably trauma, myocardial infarction (MI) and acute stroke [2]. Ambulance-based paramedics have a critical role providing rapid pre-hospital assessment, triage and expediting access to these emergency treatments. However, a review of 21 clinical handover studies raised concerns about the quality of communication and information exchange between pre-hospital and hospital staff, particularly when under time pressure due to service demands [2–4]. Structured handover frameworks have been shown to improve the accuracy and speed of information transfer between paramedic and emergency department (ED) staff, [5] but it is unclear whether condition-specific versions could further improve care and health outcomes for time-critical conditions.

In clinically complex situations, patient outcomes might be improved further if paramedics and/or emergency medical technicians continued to contribute actively towards care alongside the emergency hospital team for a limited time after handover, such as assisting with airway management or intravenous access. Following handover of standard clinical information, paramedics do not routinely continue to be part of patient’s ongoing care in the ED, coronary care, stroke unit or other appropriate specialist treatment facility in secondary care. However, they might possess relevant skills at a time when the hospital team can be subject to competing demands, which could contribute further towards faster access to emergency treatments. Achieving optimal health outcomes may require development of enhanced clinical roles which transgress traditional boundaries.

In order to summarise the current evidence describing the impact of enhanced paramedic processing of emergency conditions with time-dependent treatment outcomes (i.e. trauma, stroke and MI), we undertook a review of literature reporting the clinical effectiveness of (i) structured paramedic/emergency medical technician handovers to emergency hospital teams, and (ii) paramedics/emergency medical technicians or ambulance technicians continuing to contribute actively towards care alongside the emergency hospital team after handover (i.e. an enhanced/expanded role across the threshold of secondary care).

In order to provide additional context in terms of process/operational data relevant to time-critical clinical conditions that are the current focus of service re-structuring to facilitate care pathways to improve health outcomes, a secondary objective was to categorize and narratively describe relevant studies which did not meet the full inclusion criteria but explored either: (i) development and evaluation of novel structured handover tools or protocols; (ii) protocols or enhanced skills to improve handover; or (iii) protocols or enhanced skills leading to a change in in-hospital transfer location.

## Methods

The review process adhered to a published protocol [6] and reporting guidelines of the Preferred Reporting Items for Systematic Reviews and Meta-analyses (PRISMA) statement [7].

### Study designs

Experimental, quasi-experimental and observational research studies, including qualitative and mixed methods studies were eligible for inclusion. Eligible studies involved paramedics, emergency medical technicians (EMTs) or ambulance technicians (including the armed forces) that reported on the development, evaluation or implementation of (i) novel structured handovers to hospital-based physicians for acute stroke, acute MI or trauma patients; or (ii) new processes at handover or post-handover clinical activity that actively contributed towards a patient’s care alongside the emergency hospital team for a limited time after handover in the secondary care setting for acute stroke, acute MI or trauma. Trauma patients were defined as those with any life-threatening injury requiring urgent treatment within 24 h. Eligible studies were also required to assess changes in health outcomes (e.g. survival, quality of life or functional status at 24 h or discharge).

Studies were excluded if the focus was on pre-hospital activity only (e.g. telephone communication, implementation of Advanced Cardiac Life Support algorithms), protocol driven ambulance redirection (e.g. to a different hospital or specialist hub), non-clinical activity (e.g. evaluation of electronic record systems) or involved transportation with an accompanying physician (i.e. patient care was medically-driven from the outset). Paramedics/emergency medical technicians with extended community roles designed to reduce hospital admissions and those already based in hospital (i.e. working routinely in an emergency department) were also excluded if they did not involve an interaction with a hospital for avoiding or facilitating emergency admissions.

### Search strategy

A structured search strategy (MeSH terms and keywords) designed by an experienced information scientist (SR) was applied to bibliographic databases (MEDLINE, PubMed, HMIC - Health Management Information Consortium, Cochrane HTA, Cochrane Central, EMBASE, ASSIA and PsycInfo. Main search terms included ambulance, EMIS, paramedic, pre-hospital, myocardial infarction, stroke and trauma (see Appendix 1 for an example of the full search strategy in MEDLINE). The design, treatments and professional roles within routine emergency medical services have evolved significantly since the 1980s; therefore the search was restricted to studies published from Jan 1990 to Sep 2016. No restrictions were placed on country of origin, but searches were restricted to abstracts published in English. Hand-searching of reference lists and citation searching of eligible studies was also undertaken (including relevant reviews identified by the search strategy). Grey literature was identified from contact with content experts (NASMED, College of Paramedics).

### Study selection, data extraction and assessment of methodological quality

Two reviewers (DF and RF) independently assessed the titles and abstracts retrieved via the search strategy (stage 1) and independently assessed the retained full text studies using a study selection form (Appendix 2). Disagreements at the full text stage were resolved via discussion or by consulting with a third member of the review team (CP).

Two authors independently captured information on study characteristics (study design, participants, context, any new care processes undertaken by paramedics/EMTs at handover/post-handover and outcomes) and assessed methodological quality according to the frameworks developed by the Cochrane Collaboration [8, 9].

### Data synthesis

A high degree of heterogeneity between study designs and outcomes was expected, therefore no sensitivity analyses were planned, and a narrative approach was used to synthesise the findings of included studies.

## Results

**Fig. 1:**
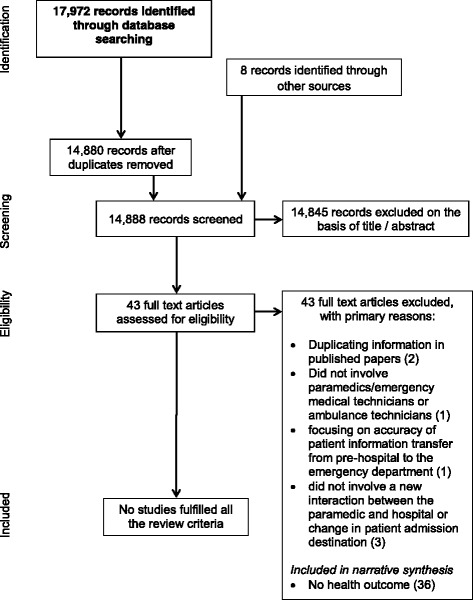
PRISMA flow diagram of the process used to identify studies

A total of 17,972 hits were generated by the search strategy (Fig. 1). Of the 43 full text articles identified following initial screening, seven were excluded for not involving paramedics/emergency medical technicians or ambulance technicians [10], focusing on accuracy of patient information transfer from pre-hospital to the ED [11], duplicating information [12, 13] in published papers [5, 14] or did not involve a new interaction between the paramedic and hospital or change in patient admission destination [14–16]. Although relevant as interventions that could be evaluated for improving health outcomes, none of the remaining 36 articles fulfilled all of the review criteria, in particular omission of an evaluation of health outcomes in the context of stroke, MI or trauma. These 36 studies have been summarised into three categories for narrative synthesis (Table 1): structured handover tools/protocols, protocols and enhanced skills to improve handover, and protocols or enhanced skills leading to a change in in-hospital transfer location.

**Table 1:** Summary of shortlisted studies

Author, publication year	Country	Design	Stroke, MI or trauma	Objectives and methods	Key findings
STRUCTURED HANDOVER TOOLS/PROTOCOLS					
Evans et al. [18]	Australia	Grounded Theory	Trauma	Qualitative study of paramedics and trauma teams to explore the utility of the MIST (M - Mechanism of injury, I - Injuries sustained, S - Signs and T - Treatment and trends in the vital signs) template and other aspects related to quality handover processes (develop a minimum dataset to assist paramedics; features of effective/ineffective handover; feasibility of advanced data transmission; and optimal mode of data display in trauma bays)	Study authors’ concluded that there is support for the adoption and further evaluation of a structured handover template. Quality handovers involved information that was vital, succinctly delivered and structured to inform immediate treatment. Pre-alert information conveyed by paramedics was considered important
Iedema et al. [5]	Australia	Video-reflexive ethnography	Trauma and non-trauma	Novel ambulance to emergency department handover protocol: IMIST-AMBO: Identification of the patient, Mechanism/medical complaint, Injuries/information relative to the complaint, Signs, vitals and Glasgow Coma Scale, Treatment and trends/response to treatment, Allergies, Medications, Background history and Other (social) information	IMIST-AMBO showed promise for improving handover communication between emergency medical services and emergency department physicians (greater volume of information per handover that was more consistently ordered; fewer questions from emergency department staff; shorter handover duration; and fewer repetitions by paramedics and emergency department staff)
Dojmi Di Delupis et al. [17]	Italy	Mixed methods	None	Communication between pre-hospital/hospital providers using analysis of simulated sessions, survey of triage nurses and expert focus groups led to the development of ISBAR (Identification, Situation, Background, Assessment and Request)	The ISBAR did not result in improved communication during handovers to hospital staff as assessed by micro-simulations
Ebben et al. [23]	The Netherlands	Prospective pre-test post-test design	None	Evaluated the effectiveness of an e-learning tool for improving adherence to a guideline for structuring pre-hospital notification and handover: • DeMIST: Demographics, Mechanism of Injury/illness, Injury or Illness found or suspected, Signs, Treatment given) The e-learning tool was designed with five components (1) knowledge about the DeMIST model and handover process, (2) skills to use the DeMIST model; and (3) motivation to use the DeMIST model	The authors noted a high baseline adherence rate to usage and correct sequence of the DeMIST model. However, the DeMIST e-learning program did not improve adherence to the handover guideline (no statistically significant changes in numbers of handovers with the DeMIST model or numbers of handovers consistent with the sequence of the DeMIST model
Meisel et al. [19]	USA	Qualitative study using focus group methods	Trauma	Qualitative study of professional, structural and interpersonal factors influencing handovers between emergency medical services care and emergency department, but did not evaluate a novel structured handover process	Findings suggested that increasing emergency medical services interactions with emergency physicians, standardising patient handovers and inter-professional learning would improve handovers for high-risk emergency medical services to emergency department
Von Cannon & Porcelli 2016 [24]	USA	Quality improvement project	Stroke	Evaluated the impact of a collaborative project between a hospital and an EMS agency, which aimed to improve the CODE Stroke process. The quality improvement project involved: • EMS crews giving advance notification of suspected stroke cases • EMS crews transporting patients directly to CT scanner in the emergency room, including providing assistance with the transfer • Feedback on patient treatment (t-PA) was provided to EMS crews along with dispatch to scene times, and dispatch to t-PA parameters and patient outcomes, which included a letter of commendation from their supervisor	The project reported an improvement in door to CT scan times: • mean 27 min in the year [2013] before the project • mean of 20 min in 2014 (one year after the project began) During 2014, the authors reported a stroke identification accuracy rate of 89%; and a median door to t-PA time of 40 min.
PROTOCOLS AND ENHANCED SKILLS TO IMPROVE HANDOVER					
Foster et al. [46]	USA	Prospective observational study	Myocardial infarction	Prehospital recognition of acute myocardial infarction undertaken by trained hospital-based nurses and paramedic advanced life support providers	Hospital-based nurses and paramedic advanced life support providers can successfully be trained to evaluate a prehospital electrocardiogram for presence of acute myocardial infarction with accuracy.
O’Connor & Megargel 1994 [51]	USA	Before and after study	Myocardial infarction and trauma	Evaluated the impact of feedback on paramedic skills (charting, resuscitation rates from cardiac arrest, endotracheal intubation success rates and trauma scene times	Quality improvement feedback can improve charting for endotracheal intubation and reduce trauma scene times (but not resuscitation rates from cardiac arrest or endotracheal intubation success rates)
Zempsky et al. [47]	USA	Cross sectional survey	None	Evaluated paramedics who were employed as allied health care professionals (based in the paediatric emergency department as assistive personnel). No defined clinical condition other than paediatric care	Paramedics function successfully in the emergency department as members of the paediatric emergency department care team (and this may be cost-effective adjunct to nursing support)
Scott et al. [20]	USA	Uncontrolled before and after design	Trauma	Evaluated a web-based educational intervention targeting communication skills of paramedics during handover of trauma patients to emergency department clinicians	Web-based intervention failed to show a statistically significant impact on amount of clinical information recalled by physicians during handover of trauma patients.
Mason et al. [43]	UK, England	Mixed methods	None	Evaluated the appropriateness, satisfaction and cost-effectiveness of an emergency care practitioner role in a primary care practitioner led out of hrs service or nurse-led walk in centre.	Care delivered by emergency care practitioners appeared to reduce emergency hospital admissions
Campbell et al. [45]	Canada	Retrospective observational study	None	Advanced care practitioner role - based in hospital setting (and not focused on stroke, myocardial infarction or trauma)	Procedural sedation and analgesia conducted in the emergency department by trained paramedics is not associated with significant number of adverse events (only one was recorded)
Ranchord et al. [42]	New Zealand	Retrospective observational study	Myocardial infarction	Evaluated the impact of pre-hospital electrocardiogram for myocardial infarction patients, and following a decision made by a physician, a paramedic administered thrombolysis	Prehospital paramedic-administered thrombolysis was deemed to be safe and reduced time to treatment and heart failure
Chan et al. [21]	Australia	Uncontrolled before and after study	None	Focused on medication reconciliation (and not focused on stroke, myocardial infarction or trauma); i.e., paramedics were asked to bring patients’ own medication to the emergency department	Patients’ own medication was brought into emergency department more frequently and prescribing errors reduced
Waßmer et al. [22]	Germany	Uncontrolled before and after study	None	Evaluated a simple training intervention to improve communication in a rescue teams and handover to emergency department physicians using simulated emergency operations	The simple training intervention resulted in better structured communication between teams and handover of paramedics (frequency of negative communication events decreased from 3.9 per scenario before training to 1.8 after training)
Mason et al. [44]	UK, England	Quasi-experimental study	None	Evaluated the impact of the emergency care practitioner role (generic practitioner with a nursing or paramedic background) on patient pathways based in different emergency care settings (i.e., paramedics did not transport patients and were based in settings such as an urgent care centre)	Impact of emergency care practitioners is greatest when the operate in mobile settings when care is taken to the patient
Jensen et al. [41]	Canada	Health care failure mode and effect analysis	Myocardial infarction	Mapping using hazard analysis of pre-hospital treatment of ST-segment-elevation myocardial infarction patients with fibrinolytics by paramedics	ST-segment-elevation myocardial infarction calls in which paramedics administer fibrinolytics is a complex process with many steps, but relatively few were hazardous to patient care or safety
Landman et al. [49]	USA	Qualitative study	Myocardial infarction	Qualitative study exploring collaborations between emergency medical services and hospitals in the care of hospitalised acute myocardial infarction patients	Relationships between emergency medical services and acute myocardial infarction teams differed between high and low performing hospitals – high performers described multi-faceted strategies to support collaboration with emergency medical services personnel
Ryan et al. [40]	Canada	Retrospective observational study	Myocardial infarction	Aim was to investigate the proportion of ST-segment-elevation myocardial infarction patients who had clinically important events or received advanced paramedic care that was delivered in the pre-hospital period (paramedic had no direct involvement in patient care or treatment in hospital other than direct transportation to percutaneous coronary intervention laboratory)	Clinically important events (several patients needed cardiopulmonary resuscitation or defibrillation) and advanced care treatment are common in ST-segment-elevation myocardial infarction patients (e.g., administration of morphine or atropine) undergoing pre-hospital transfer or intra-facility transfer to a percutaneous coronary intervention centre
Todt et al. [25]	Sweden	Multi-stage action research study	Myocardial infarction	Paramedics conducted prehospital electrocardiogram recording for suspected ST-segment-elevation myocardial infarction patients with pre-notification to the coronary care unit	First medical contact to patient artery and catheterisation laboratory decreased by 6 and 12 mins respectively
Choi et al. [50]	USA	Uncontrolled before and after study	Stroke	A hospital-directed feedback to emergency medical services in terms of compliance with state protocols for pre-hospital assessment of stroke	Feedback improved compliance with protocols
von Vopelius-Feldt & Benger [48]	UK, England	Cross-sectional survey	None	Survey of ambulance services about the use and role of clinical care paramedics (not individual paramedics) and was not focused on stroke, acute myocardial infarction or trauma	There were variations in training, competencies and working patterns of clinical care paramedics across England
PROTOCOLS OR ENHANCED SKILLS LEADING TO A CHANGE IN IN-HOSPITAL TRANSFER LOCATION					
Dorsch et al. [31]	UK, England	Prospective observational study	Myocardial infarction	Pre-hospital diagnosis of ST-segment-elevation myocardial infarction by paramedics and direct transfer to catheterisation laboratory for primary percutaneous coronary intervention	The 90 min target for door to balloon time was achieved in 94% of direct admissions compared to 29% referred from the emergency room
Dieker et al. [30]	Netherlands	Retrospective observational study	Myocardial infarction	Pre-hospital diagnosis of ST-segment-elevation myocardial infarction by paramedics and direct transfer to an intervention centre with pre-hospital notification of the catheterisation laboratory	The protocol more than tripled the proportion of patients treated within 90 mins
Pathak et al. [26]	Australia	Retrospective observational study	Myocardial infarction	Evaluated the impact of pre-hospital electrocardiogram for ST-segment-elevation myocardial infarction patients and emergency department activation of the primary percutaneous coronary intervention team (paramedic had no direct involvement in patient care or treatment in hospital other than direct transportation to catheterisation laboratory)	Pathway (pre-hospital diagnosis of ST-segment-elevation myocardial infarction and direct transfer to catheterisation laboratory) significantly reduced door to balloon time.
Rostykus et al. [37]	USA	Retrospective observational study	Myocardial infarction	Evaluated the impact of pre-hospital emergency department activation of catheterisation laboratory by paramedics on mortality compared with referring emergency department activations	Mortality rates for ST-segment-elevation myocardial infarction patients in hospital were not significantly different between pre-hospital emergency department and referring emergency department activations
Larsson & Holgers 2011 [35]	Sweden	Retrospective observational study	Trauma (hip fracture)	Evaluated the impact of nurse paramedic assessment of hip fracture and direct transfer to radiology (paramedic had no direct involvement in patient care or treatment in hospital other than transportation to radiology and delivery of blood test results to laboratory)	Study suggests that ‘fast-track’ care can minimise complications for patients with suspected hip fracture and overall length of care
Dewhurst & McComb 2012 [29]	England, UK	Retrospective observational study	Complete heart block	Direct transfer of patients with complete heart block by ambulance service to a pacing centre, for urgent pacing	Direct transfer from the ambulance service was appropriate (may reduce complications and length of stay)
Huang et al. [33]	USA	Retrospective observational study	Myocardial infarction	Direct transfer to catheterisation laboratory of ST-segment-elevation myocardial infarction patients by helicopter paramedics with pre-alert and pre-hospital treatment	Study demonstrated feasibility of emergency medical services-activated ST-segment-elevation myocardial infarction protocol over long distances with good reperfusion times
Birkemeyer et al. [38]	Germany	Retrospective observational study	Myocardial infarction	Comparison of mean door to hospital and mean door to primary percutaneous coronary intervention times in two myocardial infarction network registries: one with (*n* = 322 patients) and one without (*n* = 494) bypass of emergency rooms (direct transfer to catheterisation laboratory)	Mean time delay between onset and arrival at hospital was statistically significant shorter in the network with direct transfer to catheterisation laboratory (196 min versus 257 mins in the network that did not) Mean door to primary percutaneous coronary intervention time was statistically significantly lower in the network with direct transfer to catheterisation laboratory (15 mins) than the network that did not (21 mins)
Colleran et al. [28]	Ireland	Prospective observational study	Myocardial infarction	Evaluated the impact of electronic transmission of prehospital assessment of ST-segment-elevation myocardial infarction patients by paramedics and direct transfer to catheterisation laboratory	Electronic electrocardiogram transmission did not reduce rates of inappropriate catheterisation laboratory activation or reduce door to balloon times
Kendall et al. [34]	UK, England	Retrospective observational study	Stroke	Paramedics transported patients with suspected stroke directly to computerised tomography scanner (paramedic had no direct involvement in patient care or treatment in hospital other than transportation to computerised tomography)	The direct to computerised tomography scan pathway successfully reduced delays to thrombolysis treatment (call to door and computerised tomography to needle times were not improved)
Meretoja et al. [36]	Australia	Prospective observational study	Stroke	Paramedics pre-notified stroke teams with patient details and transported patients directly to computerised tomography scanner (paramedic had no direct involvement in patient care or treatment in hospital other than transportation to computerised tomography).	The enhanced stroke thrombolysis protocol was demonstrated to be transferable to the Australian healthcare setting
Binning et al. [27]	USA	Retrospective observational study	Stroke	Pre-hospital alert by emergency medical services for suspected stroke that bypasses the emergency department (straight to computerised tomography scan)	Decreased door to computerised tomography scan and door to needle times
Farshid et al. [32]	Australia	Prospective observational study	Myocardial infarction	Pre-hospital diagnosis of ST-segment-elevation myocardial infarction by paramedics and direct transfer to catheterisation laboratory	Ambulance diagnosis of ST-segment-elevation myocardial infarction and direct transfer to catheterisation laboratory was associated with shorter treatment times and better outcomes including lower mortality
Schustereder et al. [39]	Austria	Single site before and after study	Stroke	Evaluated adherence to the Helsinki model for improving door to needle times (t-PA): 1) ambulance pre-notification with patient details alerting the stroke team 2) patients transferred directly onto the CT scan table on the ambulance stretcher 3) t-PA delivered in CT immediately after imaging.	Median (interquartile range) door to treatment with t-PA was reduced from 49.5 (35–95) minutes in the first month of the observation period to 29 (8.5–64.5) minutes in the fifth month

Seven studies described approaches to improve clinical handover between paramedics/EMTs and hospital-based ED clinicians, but did not focus specifically on stroke, MI, or trauma patients or include a health outcome [5, 17–22]. For trauma patients, an uncontrolled before and after design [20] was used to evaluate the impact of a web-based, educational intervention designed to enhance paramedic verbal communication skills during handover to hospital physicians. Research associates collected recordings and made notes of patient handover conversations and then interviewed physicians to assess their recall of details provided by paramedics. Overall physicians recalled 36% of paramedic verbal reports, but pre- and post-intervention levels of recall by physicians failed to reach statistical significance (33 and 38%, *p* = 0.16). In a similar study, a simple training intervention (3 h in duration) consisting of five rules of communication, simulated case scenarios and a handover protocol (inventory, medical history, clinical findings and actions) reported that the frequency of negative communication events decreased from 3.9 per scenario before training to 1.8 after training [22]. A focus group study [19] identified the following professional, structural and interpersonal factors related to improvement of trauma handovers from the perspective of paramedics (*N* = 48): direct communication with ED clinician who was responsible for care of the patient; inter-disciplinary understanding and feedback to nurture a shared understanding of paramedic and ED roles; standardising selected aspects of the handover process; and use of technology to facilitate information exchange. However there was no evaluation of these principles in practice.

Four studies reported on the development of new generic protocols/checklists to improve handover quality [5, 17, 18, 23]. In a grounded theory study [18] ten paramedics and 17 hospital-based trauma clinicians were interviewed regarding adaptation of the MIST tool (Mechanism, Injury Pattern, Signs and Treatment) and highlighted the importance of concise delivery of structured information to inform immediate treatment, and hospital pre-notification. A prospective pre-test post-test study [23] evaluated the effectiveness of an e-learning tool for improving adherence to DeMIST (Demographics, Mechanism of Injury/illness, Injury or Illness found or suspected, Signs, Treatment given), but failed to show any changes in use or adherence to the sequence of this tool for structuring pre-hospital notification and handover. An Australian study used video-reflexive ethnography [5] to demonstrate the feasibility of improving handover communication between paramedics and ED staff using a standard order of information (IMST AMBO: Identification, Mechanism, Injuries, Signs, Treatment, Allergies, Medications, Background, Other) as indicated by an increase in the amount of consistently ordered information per handover, fewer clarifications from ED staff, shorter handover duration, and increased eye contact. Another observational study using video assessment was unable to show better communication from applying a structured format (ISBAR: Identification, Situation, Background, Assessment and Request) during simulated handovers [17]. This may reflect the basic content of the information conveyed and the restrictive nature of the short simulations.

One study focused specifically on the role of paramedics in relation to patient medication during handover [21], which reported a statistically significant increase in the percentage of patients’ who were reconciled with their own medication in the ED (67% at pre-intervention versus 87% post-intervention) and a reduced percentage of errors in regular medications prescribed (18.9% pre-intervention versus 8.8% post-intervention).

Sixteen studies reported on paramedic actions for trauma, stroke and MI patients that were in addition to standard handover [24–39]. They did not directly provide patient care, and the paramedic role was restricted to prehospital activation [25, 37] or transportation of patients to condition-specific specialist facilities such as catheterisation laboratory for MI patients [26, 28, 30–33, 38], radiology for acute stroke patients [24, 27, 34, 36, 39], radiology for trauma (hip fracture) patients [35], or a pacing centre for patients with complete heart block [29]. In one study paramedics were also responsible for delivery of blood test results of trauma (hip fracture) patients to the hospital laboratory [35]. The primary aim of these studies was to improve process measures, with one study reporting improved call to door times [34], two improved door to imaging times [24, 27] and eight improved door to treatment times [26, 27, 30–34, 36, 38].

Several studies reported positive findings in hospital resulting from ‘advanced paramedic’ skills but these had only been applied during the pre-hospital phase such as clinical diagnoses of ST elevation MI [30–32] and paramedic-administered morphine or atropine [40] and fibrinolytic treatment [41, 42].

Amongst the studies were descriptions of successfully implemented novel assistive paramedic roles within primary care settings/community-based walk-in centres [43, 44], but with the intention of reducing emergency admissions to the ED. Three studies reported on paramedics based in hospitals providing assistance to clinical teams by administration of procedural sedation and analgesia [45], evaluating a prehospital electrocardiogram (ECG) for presence of MI [46] and assessment of paediatric patients [47]. The range of such activities undertaken by paramedics in the UK National Health Service (NHS) was described in a survey [48], which highlighted the variation in training, competencies and working patterns, but did not identify any handover or post-handover paramedic-led interventions for trauma, stroke and MI.

Feedback to paramedics by the hospital team was considered important but was not compared against future health outcomes. 148 hospital staff involved with MI patients were interviewed about their relationship with EMS services [49] and those from high performing hospitals (upper 5% of hospitals based on 30-day standardised risk of mortality) described provision of feedback as important. Other strategies were a high level of respect for emergency medical services as valued professionals/colleagues; employing a hospital-based liaison to deliver training and facilitate communication between pre-hospital and in-hospital teams; and involvement of emergency medical services providers in care improvement initiatives. Provision of quality improvement feedback to paramedics was reported in three further studies [24, 50, 51] which resulted in better documentation for endotracheal intubation and reduced on-scene times for trauma [51]; increased adherence to pre-hospital protocols for acute stroke [50] and improved door to CT scan times for acute stroke [24].

## Discussion

We did not identify any original research describing the impact on health outcomes from condition-specific structured handover, or from extending the role of ambulance clinicians across the threshold of secondary care for trauma, stroke and MI patients. However, based upon qualitative and observational studies there are promising interventions for further evaluation which could be introduced at different points along the patient care pathway.

The role of ambulance-based paramedics across the threshold of secondary/tertiary care was limited to ‘direct transportation’ of patients to imaging facilities without further involvement in assessment or treatment, usually in an attempt to improve process measures (door to imaging and door to treatment times). Direct transportation is already a key feature of emergency care planning in the UK National Health Service for trauma, stroke and MI [2]; however there is no such policy initiative for paramedics to apply additional clinical skills during and after handover to hospital personnel. However, studies with relevance to the further development/extension of the ambulance-based paramedic role do show that there is potential utility from training paramedics to use standardised communication tools. This was the focus of a previous review [4] which also concluded that evidence favouring structured handover tools (utilising mnemonics) to improve communication during handover is lacking [4]. Consistent with our review there remains insufficient evidence to make any recommendation about condition-specific structured handovers, but if generic formats are helpful then there may be a higher impact from using tailored frameworks which communicate vital information during time-sensitive scenarios. Likewise, condition-specific feedback appears to be of value to paramedics, but is challenging to provide in a timely and structured fashion to a dispersed workforce constantly responding to high service demands.

A range of novel pre-hospital roles for paramedics with enhanced skills (diagnosis, clinical decision making and administration of treatment that was previously the responsibility of hospital physicians) have been developed and implemented successfully [43–47], with improved process outcomes and no additional risk for patients. These enhanced paramedic skills may be directly transferable to working in partnership with hospital ED clinicians across the threshold of secondary care but it was only paramedics already specifically employed to work in a hospital or community healthcare unit who had the opportunity to use the broadest range of skills e.g. as emergency care practitioners (ECP). A literature review of ECPs based at healthcare sites [52] reported benefits in terms of improved patient-reported care experience and cost savings, including a reduction in inappropriate referrals to emergency departments following review by community ECPs. Consistent with the current review, strong recommendations were not possible as reports comprised short term retrospective observational reports following recent new investment, and there were no trial evaluations of health outcome.

Given the funding pressures across the entire health care system, it would be prudent to identify how to further capitalise on enhanced paramedic competencies by developing integrated care protocols which focus on health outcomes rather than traditional professional roles and organisational boundaries. The impact of this approach upon outcomes following acute stroke is currently being assessed in the UK by the Paramedic Acute Stroke Treatment Assessment (PASTA) trial [53], which is evaluating the clinical and cost-effectiveness of an enhanced paramedic role before and after admission of patients with suspected stroke.

The main limitation of this review has been reliance upon a narrative description of studies to explore the context surrounding the primary review objective for which no direct evidence was identified. It is also possible that unpublished studies exist which report on the impact of condition-specific structured handover, or an extended role of paramedics in hospital on health outcomes for trauma, stroke and MI patients. Eligible studies may have been missed due to excluding non-English language papers and the absence of MeSH terms in the electronic databases for extended paramedic roles.

## Conclusions

Due to the nature of the studies identified, no strong recommendation can be made about changing the handover or post-admission roles of ambulance-based paramedics in hospital for patients with time-critical conditions. However, the literature identified illustrates that paramedic competencies and roles are evolving rapidly and their direct involvement in treatment of patients across the threshold of secondary care in partnership with hospital clinicians has potential to benefit health outcomes. A ‘new wave’ of paramedic research is needed to inform the design of cost-effective handover and feedback processes, and the health impact resulting from enhanced communication and inter-professional sharing of clinical skills.

## Appendix 1

Medline search strategy

1. ambulance*.ti,ab.
2. CCPs.ti,ab.
3. paramedic*.ti,ab.
4. (emergency adj3 (service* or technician* or practitioner* or dispatch* or despatch* or triage or communication?)).ti,ab.
5. EMS.ti,ab.
6. emts.ti,ab.
7. ecps.ti,ab.
8. acps.ti,ab.
9. (air adj2 rescue*).ti,ab.
10. HEMS.ti,ab.
11. out-of-hospital.ti,ab.
12. (out adj2 hospital).ti,ab.
13. pre?hospital.ti,ab.
14. tele?medic*.ti,ab.
15. (remote adj2 consult*).ti,ab.
16. tele?consult*.ti,ab.
17. tele?stroke.ti,ab.
18. or/1-17
19. exp *emergency medical services/
20. exp emergency medical technicians/
21. exp *allied health personnel/
22. exp *Transportation of patients/
23. Emergency medical service communication systems/
24. time-to-treatment/
25. exp emergency service, hospital/
26. *telemedicine/
27. *remote consultation/
28. or/19-27
29. *professional role/
30. Patient Handoff/
31. Patient Care Team/
32. (extend* adj3 (role? or skill? or scope?)).mp.
33. (transfer* adj2 patient?).mp.
34. (role? adj7 (development? or develop or developing or developed)).mp.
35. (role? adj7 impact?).mp.
36. (expand* adj3 (role? or skill? or scope? or responsibilit* or practice?)).mp.
37. ((extra or added or additional) adj4 responsibilit*).mp.
38. (paramedic* adj3 (skill? or role? or impact? or scope? or practice?)).mp.
39. (emerging adj3 (role? or skill? or scope? or responsibilit* or practice?)).mp.
40. (trends adj7 (paramedic* or role? or skill? or scope?)).mp.
41. (impact? adj9 (paramedic* or role? or skill? or scope?)).mp.
42. (integrat* adj5 (paramedic* or role? or skill? or scope?)).mp.
43. (innovati* adj5 (paramedic* or role? or skill? or scope? or practice?)).mp.
44. (patient* adj4 (handoff or handover or managment)).mp.
45. (advanced adj2 (role? or skill? or scope? or practice?)).mp.
46. (blur* adj4 (boundar* or role?)).mp.
47. (co?operati* adj5 (work or working or role? or team? or practice? or paramedic*)).mp.
48. (develop* adj4 responsibilit*).mp.
49. ((evolution or evolv*) adj3 (practice? or role?)).mp.
50. non-medical.mp.
51. ((inter?disciplin* or inter?professional or multi?disciplin* or multi?faceted) adj5 (work or working or role? or team? or staff or practice?)).mp.
52. (substitut* adj2 role?).mp.
53. (medical adj2 substitut*).mp.
54. (new adj3 role?).mp.
55. (scope adj3 practice?).mp.
56. (greater adj2 role?).mp.
57. or/29-56
58. (injury or injuries or injured).mp.
59. infarction?.mp.
60. h?emorrhage.mp.
61. stroke.mp.
62. (collision* or accident*).mp.
63. (trauma or traumas or traumatic).mp.
64. poly?trauma.mp.
65. life-threatening.mp.
66. (life adj2 threat*).mp.
67. (pre?hospital adj3 (diagnos* or managment or assessment)).mp.
68. (emergency adj2 patient*).mp.
69. (hospital adj2 door).mp.
70. door-to-needle.mp.
71. door-to-imaging.mp.
72. (emergency adj3 (scan or imaging)).mp.
73. urgent.mp.
74. pre?notif*.mp.
75. (priorit* adj2 (pre?hospital or higher or increased)).mp.
76. (immediacy adj2 (treatment or referral)).mp.
77. rapid transfer.mp.
78. (delay* or timely or urgen* or priorit* or immedia* or rapid or critical* or sever* or emergenc*).mp.
79. exp stroke/
80. critical illness/
81. exp myocardial infarction/
82. *“severity of illness index”/
83. *emergency medicine/
84. or/58-83
85. (18 or 28) and 57 and 84

## Appendix 2

Study Selection Form

**Table 2:** Study selection form

Study ID:	
1. STUDY DESIGN	Circle one response
RCT, non-randomised trial, quasi-experimental (e.g., time series), controlled before-and-after study, cohort study, case–control study, cross-sectional study or qualitative study (including mixed methods)	Yes/No/Unsure
• Exclude if case series or case study	
2. PARTICIPANTS	
Paramedics/EMTs (or ambulance technicians) in any setting clearly providing a different care process for patients admitted to hospital as an emergency. Include the armed forces but exclude air ambulance or other transportation which includes an accompanying doctor.	Yes/No/Unsure
3. INTERVENTION	
Development, evaluation or implementation of novel structured handovers or post-handover clinical activity directly related to patient care in hospital for one of the following: • acute stroke • acute MI • trauma patients Exclude non-clinical activity e.g. evaluation of electronic record systems.	Yes/No/Unsure
4. OUTCOMES:	
Quantitative assessment of change in a health outcome?	Yes/No/Unsure

Yes for 1, 2, 3 AND 4 → INCLUDE

No for 1, 2, 3 OR 4 → EXCLUDE (note reason[s] in comments box)

## Abbreviations

CT: Computerised tomography
ECG: Electrocardiogram
ED: Emergency department
EMTs: Emergency medical technicians
IMST AMBO: Identification, mechanism, injuries, signs, treatment, allergies, medications, background, other
ISBAR: Identification, situation, background, assessment and request
MI: Myocardial infarction
MIST: Mechanism, injury pattern, signs and treatment
NHS: National health service
PASTA: Paramedic acute stroke treatment assessment
PRISMA: Preferred reporting items for systematic reviews and meta-analyses
